# Epstein–Barr virus reactivation in pediatric allogeneic stem cell transplant recipients: an 11-year experience on viral load and B lymphocyte monitoring strategy

**DOI:** 10.3389/fimmu.2024.1492367

**Published:** 2024-10-25

**Authors:** Giulia Ferrando, Francesca Bagnasco, Filomena Pierri, Sara Pestarino, Gianluca Dell’Orso, Stefano Giardino, Eddi Di Marco, Maria Santaniello, Elio Castagnola, Maura Faraci

**Affiliations:** ^1^ Infectious Diseases Unit, Department of Pediatrics, Istituto di Ricovero e Cura a Carattere Scientifico (IRCCS) Istituto G. Gaslini, Genova, Italy; ^2^ Biostatistics Unit, Scientific Directorate, Istituto di Ricovero e Cura a Carattere Scientifico (IRCCS) Istituto G. Gaslini, Genova, Italy; ^3^ Hematopoietic Stem Cell Transplant Unit, Department of Hematology and Oncology, Istituto di Ricovero e Cura a Carattere Scientifico (IRCCS) Istituto G. Gaslini, Genova, Italy; ^4^ Molecular Medicine Laboratory, Istituto di Ricovero e Cura a Carattere Scientifico (IRCCS) Istituto G. Gaslini, Genova, Italy

**Keywords:** hematopoietic stem cell transplantation, viral infection, Epstein-Barr, pre-emptive therapy, rituximab, PTLD

## Abstract

**Background:**

Epstein–Barr virus (EBV) reactivation represents a frequent condition after allogeneic hematopoietic stem cell transplantation (allo-HCT) and can cause the development of a severe complication: post-transplant lymphoproliferative disease (PTLD). This retrospective study aims at investigating the incidence of EBV reactivations and analyzing the potential impact of recipient/donor-related transplant-related factors in pediatric patients. The secondary objective was to study the consequences of the approach used at our center regarding the initiation of pre-emptive therapy.

**Methods:**

This study used a retrospective evaluation of patients aged ≤25 years who received an allo-HCT at IRCCS (Istituto di Ricovero e Cura a Carattere Scientifico) Istituto Giannina Gaslini, between 2012 and 2022, with follow-up censored in July 2023. Criteria to start rituximab were as follows: a viral load ≥20,000 copies/10^5^ PBMCs or ≥10,000/10^5^ PBMCs associated with a rise in the proportion of CD 20+ lymphocytes.

**Results:**

Overall, 214 allo-HCTs were performed in 189 patients. A total of 127 (59.3%) procedures were complicated by at least one EBV reactivation, but in only one rituximab was administered. All other reactivations were characterized by viremia below reference ranges and no increase in CD20+ lymphocytes, without clinical consequences. Risk factors for EBV reactivation identified were associated with delayed immune reconstitution.

**Conclusion:**

These results could suggest the effectiveness of the approach used in providing pre-emptive therapy. The strategy adopted differs from that highlighted by other studies and allowed the reduction of the number of children who received rituximab. It has proven effective considering the low incidence rate of PTLD and reduces the risk of rituximab-related adverse events.

## Introduction

Epstein–Barr virus (EBV) reactivation may occur after allogeneic hematopoietic stem cell transplantation (allo-HCT) in relation to the immunosuppression that characterizes this therapeutic approach. While most EBV reactivations are asymptomatic and do not require intervention, in 0.45% to 29% (1) of cases, reactivation can lead to post-transplant lymphoproliferative disease (PTLD). Rituximab is the first-line treatment for PTLD and can also be administered as a pre-emptive therapy to prevent the development of a full-blown PTLD. However, there is no consensus on the cutoff of EBV-DNA copies for starting pre-emptive therapy. Different thresholds proposed are 1,000 copies/mL, 10,000 copies/mL, or 40,000 copies/mL when determined in whole blood, plasma, or serum, respectively; or 1,000 copies/10^5^ peripheral blood mononuclear cells (PBMCs) (2).

In 2009, Faraci et al. (1) suggested a diagnostic approach based on the monitoring of EBV-DNA in whole blood, expressing the viral load to 10^5^ PBMCs. After a prospective study including 80 children who received allo-HCT, the authors suggested criteria to start rituximab as a viral load ≥20,000 copies/10^5^ PBMCs or ≥10,000/10^5^ PBMCs associated with a rise in the proportion of B lymphocytes (considering the percentage value of CD20+ cells in two subsequent determinations). Since then, these criteria have been used at IRCCS (Istituto di Ricovero e Cura a Carattere Scientifico) Istituto Giannina Gaslini (IGG), Genoa, Italy, for the management of children developing EBV reactivation after allo-HCT. This strategy had been adopted to reduce the use of rituximab that can cause infusion-related severe adverse events as well as delay in B-lymphocyte recovery causing persistent hypogammaglobinemia that may last even for years (3), bronchiolitis obliterans (4), and neutropenia (5, 6).

This approach (1) differs from that recently described by Marjańska et al. (7), which provides the administration of a single pre-transplant dose of rituximab in all patients, as prophylaxis, in order to reduce the number of EBV infections and the cases of PTLD in the pediatric population.

The aim of this study was to describe the incidence of EBV reactivation and PTLD after allo-HCT, and to analyze the potential impact of recipient/donor-related or transplant-related factors in a pediatric setting.

The secondary objective was to study the consequences of the approach used at our center presenting the results of an 11-year study on the effectiveness of using EBV copies/10^5^ PBMCs combined with changes in CD20+ lymphocyte proportions as criteria for administering rituximab as pre-emptive therapy.

## Patients and methods

We retrospectively evaluated all patients aged ≤25 years who received an allo-HCT at IGG between 2012 and 2022 with follow-up censored in July 2023. The EBV-DNAemia in blood samples was monitored twice a week by polymerase chain reaction (EBV R gene TM quantification kit from Argene-Biomarieux company, with a sensitivity limit of 182 copies/mL and an upper limit of 10^7^ copies/mL). Monitoring began at the start of the conditioning regimen and continued until day +100 post-transplant, regardless of donor and recipient serological status, or for a longer duration in patients with delayed immune recovery. EBV reactivations occurring before this monitoring period were excluded. All patients received acyclovir prophylaxis (30 mg/kg/day iv) post allo-HCT until day +100 or until cessation of immunosuppression for prevention of Herpes simplex virus 1–2 reactivation. Our study protocol was approved by the Institutional Ethics Committee on 11 November 2019 (number 273/4668). Written informed consent was obtained from the patient or her/his legal representative.

### Statistical analysis

Descriptive statistics were reported in terms of absolute frequencies and percentage for categorical data, and the Pearson’s chi-square test or Fisher’s exact test, if appropriate, was applied to compare proportions. Continuous data were described in terms of median values, range, and interquartile range (IQR) due to their non-normal (Gaussian) distribution. Accordingly, comparisons between groups were made by the non-parametric Mann–Whitney *U*-test.

The counting process approach was applied to consider that any patient could have received more than one allo-HCT.

The association between binary outcome variable (EBV reactivation) and independent variables (demographic and allo-HCT features) was assessed by a standard logistic regression model and reported in terms of the odds ratio (OR) and 95% confidence interval (CI) with the robust estimator of variance allowing for intra-group (intra-patient) correlation. The likelihood ratio (LR) test was used to measure the effect of each predictor, and variables that were significantly associated with the study outcome were identified using a backward selection procedure.

All tests were two-tailed and a *p*-value <0.05 was considered statistically significant. All analyses were performed using Stata (StataCorp. Stata Statistical Software, Release 18.0 College Station, TX, Stata Corporation, 2023).

## Results

During the study period, 214 allo-HCTs were performed in 189 patients. The donor was a matched unrelated or haploidentical or related donor in 86 (40.2%), 85 (39.7%), and 43 (20.1%) procedures, respectively. The source of stem-cell graft was mainly bone marrow (65.4%). In 46 haploidentical transplants with αβ/CD19 depletion, rituximab was administered the day before the infusion of peripheral stem cells, in order to prevent PTLD, as prescribed by the transplant protocol. The remaining 39 haploidentical HCTs were treated with post-transplant cyclophosphamide as GvHD prophylaxis. A total of 127 (59.3%) allo-HCTs were complicated by at least one EBV reactivation but only one (0.8% of all reactivation episodes) patient received rituximab for PTLD therapy.

The univariate analyses of potential risk factors for EBV reactivation (**Table 1**) showed that non-malignant diseases are protective for the development of EBV reactivation (*p* < 0.001). This analysis showed a significant association between EBV reactivation and use of steroids (>2 mg/kg/day) (*p* = 0.009), acute (a-) or chronic (c-) GvHD (*p* = 0.04 and *p* = 0.003, respectively), and donor type (*p* = 0.012). These results were partially confirmed by the logistic regression model (**Table 2**): allo-HCTs performed for a non-malignant disease had lower risk of EBV reactivation with respect to allo-HCTs for malignant diseases, OR = 0.3, 95% CI (0.1–0.5), *p* ≤ 0.001. In contrast, HCTs complicated by cGvHD had a threefold risk of EBV reactivation with respect to HCTs not complicated by GvHD, OR = 3.1, 95% CI (1.3–7.7), *p* = 0.006. Demographic variables and the other allo-HCT features analyzed (serological risk, allo-HCT subsequent to the first, use of anti-TNF or other immunosuppressive therapy) did not emerge as factors statistically related to reactivation.

**Table 1:** The analysis of association between EBV reactivations and the characteristics of HCT.

Variables	No reactivation, *n* = 87	Yes reactivation, *n* = 127	*p*-value
**Age at HCT, years, median (IQR)**	7.8 (2.6–13.6)	8.1 (3.5–13.6)	0.752
**Sex, male, *n* (%)**	52 (59.8)	79 (62.2)	0.720
**Non-malignant diagnosis, *n* (%)**	61 (70.1)	50 (39.3)	**<0.001**
**Presence of at least one risk factor (included serological), *n* (%)**	63 (72.4)	88 (69.3)	0.623
**High serological risk (D+, R−), *n* (%)**	21 (24.1)	34 (26.8)	0.418
**Subsequent HCT, *n* (%)**	15 (17.2)	14 (11.0)	0.192
**Haplo, *n* (%)**	44 (50.6)	41 (32.3)	**0.007**
**Donor type (%)**	-	-	**0.012**
**Haplo**	44 (50.6)	41 (32.3)	-
**AD**	32 (36.8)	54 (42.5)	-
**RD**	11 (12.6)	32 (25.2)	-
**Anti-TNF, *n* (%)**	10 (11.5)	25 (19.7)	0.112
**Third-line immunosuppressive therapy, *n* (%)**	9 (10.3)	9 (7.1)	0.399
**MPD >2 mg/kg, *n* (%)**	10 (11.5)	33 (26.0)	**0.009**
**aGvHD, *n* (%)**	29/73 (39.7)	67/122 (54.9)	**0.040**
**aGvHD grade III–IV, *n* (%)**	13/29 (44.8)	42/67 (62.7)	0.104
**cGvHD, *n* (%)**	8/64 (12.5)	38/118 (32.2)	**0.003**
**cGvHD extensive, *n* (%)**	3/8 (37.5)	17/38 (44.7)	0.999

Non-malignant diagnosis = immunodeficiencies (n = 35, 31.5%), bone marrow failure (*n* = 33, 29.8%), aplastic anemia (n = 26, 23.4%), thalassemia (n = 6, 5.4%), sickle cell disease (n = 5, 4.5%), autoinflammatory diseases (n = 3, 2.7%), and metabolic diseases (n = 3, 2.7%); malignant diagnosis = lymphoblastic leukemia (n = 51, 49.5%), myeloblastic leukemia (n = 34, 33%), juvenile myelomonocytic leukemia (n = 3, 2.9%), myelodysplastic syndrome (n = 7, 6.8%), lymphoma (n = 7, 6.8%), and recurrent neuroblastoma (n = 1, 1%).

Bold values indicate the variables that were statistically significant at the analysis.

**Table 2:** Logistic regression model reported in terms of the odds ratio (OR) and 95% CI with the robust estimator of variance allowing for intra-group (intra-patient) correlation.

Variables	Odds ratio (95% CI)	p-value	
**Non-malignant diagnosis vs. malignant**	0.3 (0.1–0.5)	<0.001	
**cGvHD, yes vs. no**	3.1 (1.3–7.7)	0.006	

The patient who was treated with rituximab was an 8-year-old girl with aplastic anemia. She received three allo-HCTs (two from matched unrelated donors followed by graft failure and one haploidentical PTCy). At day +55 after the third procedure, she presented fever and CT scan showed multiple thoracic and abdominal lymphadenopathy and EBV copies raised to 63,418/10^5^ PBMCs combined with a rise in B lymphocytes proportion from 0% to 12% (absolute number of CD 20+ lymphocytes: 105/mmc), while receiving methylprednisolone (3.5 mg/kg/day), extracorporeal photopheresis, and anti-TNF for severe intestinal and cutaneous GvHD (global grade 4). After the infusion of rituximab (375 mg/m^2^), her conditions improved and EBV copies decreased to negativity. She was discharged on day +86, in the absence of clinical signs of GvHD or PTLD. Her last follow-up was in February 2023 (5-year after the first allo-HCT).

All other reactivations were observed, in the first post-transplant year, and were characterized by viremia values below reference ranges and no increase in CD20+ lymphocytes. No one else developed PTLD or end-organ diseases such as encephalitis/myelitis, pneumonitis, or hepatitis related to the viral infection. No patients required EBV-antiviral lymphocytes. At the last patients’ visit [median follow-up of 3.1 (IQR 1.2–5.5) years], a total of 48 deaths (25.4%) were reported in 189 patients; 35/189 (18.5%) were transplant-related (TRM) and 13/189 (6.9%) were defined as relapse related (RRM). In the TRM group, 14.3% (5/35) were related to viral infections and 4 of them were caused by adenovirus. Moreover, one patient died from herpes virus 6 infection. Of note, no deaths directly related to EBV have been observed. Furthermore, 7/35 (20.0%) patients died because of other infectious complications, related to invasive fungal diseases or bacterial infections. An additional 10 deaths (28.6%) were due to GvHD, 9 (25.7%) died due to toxicity, 2 (5.7%) died due to graft failure, 1 patient (1/35) died because of a hemorrhage, and 1 (1/35) died due to the consequence of a thrombotic microangiopathy.

Among RRM, 12/13 deaths were caused by relapse of the malignant underlying disease (12/101, 11.9%, considering only patients with a malignant diagnosis) while 1 patient died because of progression of the primary disease.

Overall cumulative survival was 82.9% (95% CI 76.7–87.6) after 1 year since HCT, 72.7% (95% CI 65–78.9) at 5 years, and 69.7% (95% CI 60.1–77.5) at 10 years.

## Discussion

In this study, we analyzed the effectiveness of using EBV copies/10^5^ PBMCs combined with changes in CD20+ proportions as criteria for administering rituximab as pre-emptive therapy for PTLD (1). Our findings confirmed that with this approach, less than 1% of post-allo-HCT EBV reactivations required rituximab infusion for the pre-emptive treatment of PTLD.

All risk factors for EBV reactivation identified in our analysis were associated with delayed immune reconstitution, which results in the loss of immune surveillance and allows the opportunistic outgrowth of EBV-infected cells. Additionally, the use of an alternative donor (AD) emerged as a significant risk factor, likely due to minor histocompatibility mismatches between donor and recipient, necessitating more intensive immunosuppression, such as ATG to prevent GvHD. In contrast to historical consensus, which suggests that patients undergoing haploidentical transplants have an increased risk of EBV reactivation (8), our cohort of haploidentical recipients experienced fewer reactivations. This finding is similar to the results of a study from Düver et al. (9), who demonstrated that the lowest risk of EBV infection was observed in haploidentical HCTs, attributing this reduced risk to the effective B-cell depletion commonly used in these patients. Moreover, in our cohort, in case of αβ/CD19-depleted HCT, rituximab was administered the day before stem cell infusion, in order to prevent PTLD. On the field of haplo-PTCy-HCT, a protective role could be exercised by the lysis of EBV-infected lymphocytes with relative sparing of memory cells. In fact, most studies (10, 11), ascribe EBV reactivation and the associated PTLD to donor-derived EBV with an increased risk in case of seronegative recipient, even if this correlation did not emerge from our statistical analysis.

These results are improved when compared with our previous report (1), when we observed 12 cases out of 87 allo-HCTs (13.8%), performed between 2004 and 2008, exceeding the viremia cutoff of 20, 000 copies/10^5^ PBMCs, with 75% (*n* = 9) requiring rituximab therapy and one developing full-blown PTLD, successfully treated with chemotherapy, rituximab, and cellular therapy. **Table 3** reports differences in patients’ characteristics in the two study periods. Remarkably, in the second period, haploidentical HCTs were performed, while the proportion of cord blood as a source of stem cells was reduced by nearly 50%. In spite of an increase in the number of transplants considered at risk of EBV reactivation (2, 8), the absolute number of PTLDs requiring rituximab remained the same (*n* = 1), leading to an impressive reduction of the proportion of allo-HCTs with clinically significant reactivation. These differences could be explained by less toxic conditioning regimens, lower incidence of GvHD, and relative lower immunosuppression.

**Table 3:** Characteristics of the HCTs in the two cohorts of allogeneic HCT.

Study group	2004–2008 *N* = 87	2012–2022 *N* = 214
Age at HCT, years, median (IQR)	7 (2.3–11)	7.9 (3.3–13.6)
Donor type, *n* (%)	-	-
AD	70 (80)	86 (40.2)
HAPLO αβ/CD19 depleted	-	46 (21.5)
RD	17 (20)	43 (20.1)
HAPLO PTCy	-	39 (18.2)
Source of stem-cell graft, *n* (%)	-	-
BM	67 (78)	140 (65.4)
PBSC	10 (11)	63 (29.4)
CB	10 (11)	11 (5.2)
Conditioning regimen, *n* (%)	-	-
MAC	59 (68)	157 (73.4)
RIC	28 (32)	57 (26.6)

HCT, hematopoietic cell transplantation; D, donor; R, recipient; AD, alternative donor; Haplo, haploidentical related donor; PTCy, post-transplant cyclophosphamide; RD, related donor; Anti-TNF, anti-tumor necrosis factor; MPD, methylprednisolone; aGvHD, acute graft versus host disease; cGvHD, chronic graft versus host disease; BM, bone marrow; PBSC, peripheral blood stem cell; CB, cord blood; MAC, myeloablative conditioning regimen; RIC, reduced intensity conditioning regimen.

**Figure 1:**
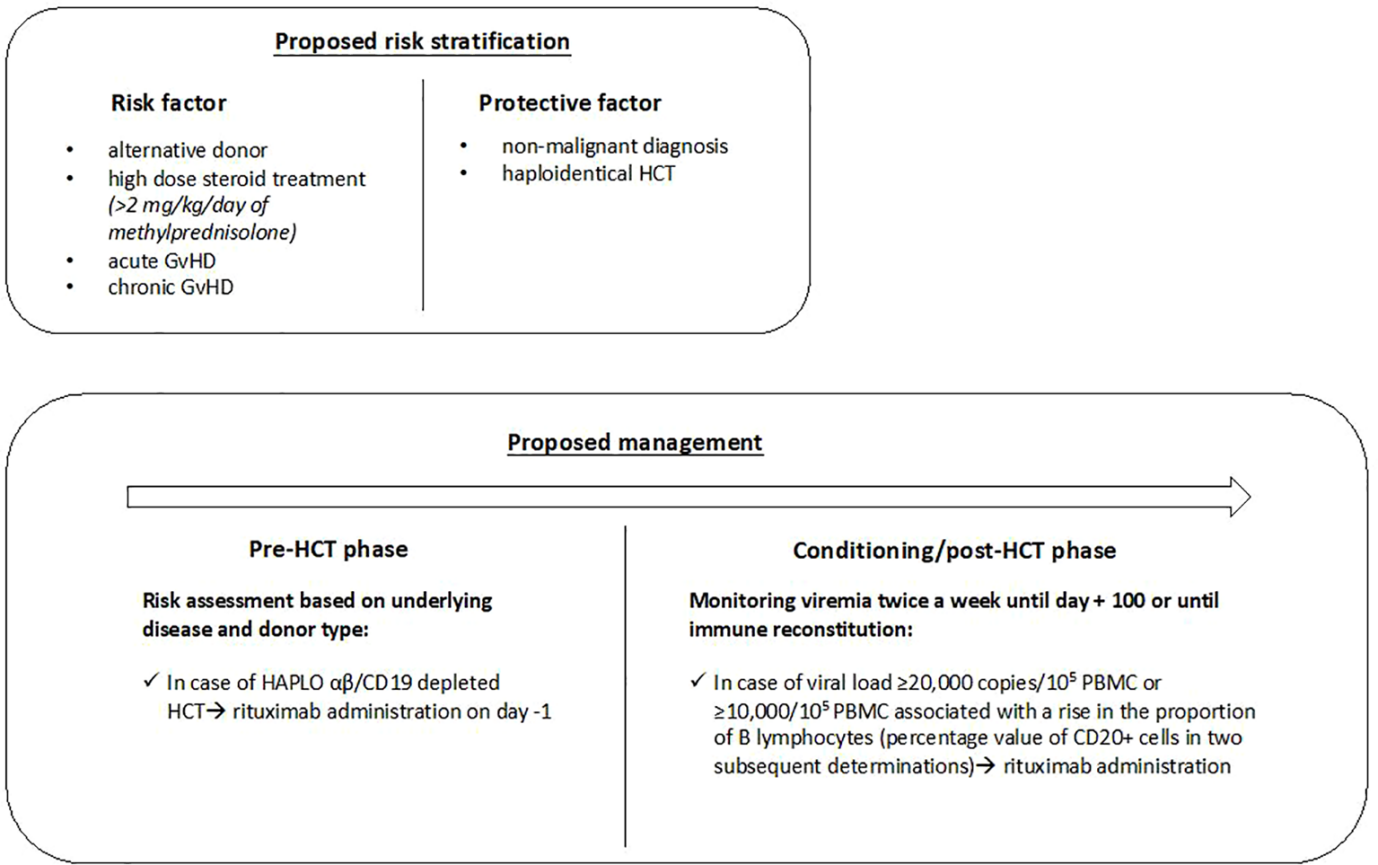
The proposed risk stratification and management in pediatric HCT recipients. HCT, hematopoietic cell transplantation; GvHD, Graft versus Host Disease; Haplo, haploidentical related donor; PBMC, peripheral blood mononuclear cell.

Based on these results, we defined risk factors and categories shown in **Figure 1** , which could be useful to guide the use of rituximab as pre-emptive therapy.

This study supports, on a larger scale, our previous observation (1) and the effectiveness of the approach used in providing pre-emptive therapy. This strategy, which could be defined more “wait-and-see,” is based on EBV viral load surveillance, CD20+ lymphocytes monitoring, and a high level of clinical suspicion. It differs from that highlighted by other studies (2, 7), and allowed us to reduce the number of children receiving pre-emptive rituximab. It has proven effective considering the low incidence of PTLD and reduces the risk of early and late rituximab-related adverse events. Furthermore, considering the low rate of PTLD and treated patients, one might consider continuing twice-weekly viremia monitoring only in patients with risk factors for EBV reactivation. Additional studies are needed to validate this approach.

## Data Availability

The raw data supporting the conclusions of this article will be made available by the authors, without undue reservation.
